# Analysis of Blood‐Brain Barrier Endothelial Junction Proteins in the 3xTg‐AD Model of Alzheimer's Disease

**DOI:** 10.1002/alz70855_102382

**Published:** 2025-12-23

**Authors:** Karen Michelle Delgado‐Minjares, Luis Oskar Soto‐Rojas, Rubén Gerardo Contreras‐Patiño, Sofía Díaz‐Miranda, Catalina Flores‐Maldonado

**Affiliations:** ^1^ Centro de Investigación y de estudios Avanzados del Instituto Politécnico Nacional, Ciudad de México, DF, Mexico; ^2^ Facultad de Estudios Superiores Iztacala, Universidad Nacional Autónoma de México, Ciudad de México, DF, Mexico; ^3^ Facultad de Estudios Superiores Iztacala, Universidad Nacional Autónoma de México, Carrera de Médico Cirujano, MEXICO CITY, EM, Mexico; ^4^ Instituto de Neurobiología, Universidad Nacional Autónoma de México, Querétaro, QA, Mexico

## Abstract

**Background:**

Alzheimer´s disease (AD) is the most prevalent form of dementia worldwide and progresses through three stages (Alzheimer's continuum): presymptomatic, mild cognitive impairment, and dementia. Pathological protein aggregates, including tau and amyloid beta (Aβ), are present throughout the disease continuum. Aβ accumulation in cerebral microvasculature leads to blood‐brain barrier (BBB) dysfunction. The BBB is a highly selective structure that regulates the transport of substances between the blood and the brain parenchyma. Endothelial junction proteins control paracellular transport at the BBB, and their levels are reduced in late‐stage AD, increasing BBB permeability. However, their role in other stages remains unexplored. Therefore, the objective of this project was to evaluate the expression level of the endothelial junction proteins (claudin‐1, ‐3 and ‐5, occludin, and VE‐cadherin) in asymptomatic, early and late stages of AD using the 3xTg‐AD murine model.

**Method:**

A total of 32 male mice (16 non‐transgenic and 16 3xTg‐AD) aged 3, 6, 12, and 16 months were analyzed (*n* = 4 per group). Capillary brain proteins were isolated, and the expression levels were determined using the western blot technique.

**Result:**

In the 3xTg‐AD mice at asymptomatic and early stages, claudin‐1, ‐3, and ‐5 expression levels increased, while occludin decreased, compared to the non‐transgenic mice. Meanwhile, there are no significant changes in the last stages of the 3xTg‐AD model.

**Conclusion:**

Our findings suggest that in the asymptomatic and early stages of the murine model of AD, the BBB increased the production of claudin‐1, ‐3 and ‐5 proteins, to compensate for the loss of the other junction proteins. This insight provides a foundation for understanding BBB dynamics in AD progression and highlights potential targets for early therapeutic intervention.

